# Strategies to facilitate the development of uncloned or cloned infectious full-length viral cDNAs: *Apple chlorotic leaf spot virus *as a case study

**DOI:** 10.1186/1743-422X-8-488

**Published:** 2011-10-31

**Authors:** Fater Youssef, Armelle Marais, Chantal Faure, Pascal Gentit, Thierry Candresse

**Affiliations:** 1 Equipe de Virologie, INRA, UMR 1332 Biologie du Fruit et Pathologie, BP81, 33883 Villenave d'Ornon cedex, France; 2 Equipe de Virologie, Université de Bordeaux, UMR 1332 Biologie du Fruit et Pathologie, BP81, 33883 Villenave d'Ornon cedex, France; 3 Laboratoire de Virologie, Ctifl, Centre de Lanxade, 24130 La Force, France

## Abstract

**Background:**

Approaches to simplify and streamline the construction of full-length infectious cDNA clones (FL-cDNAs) are needed. Among desirable improvements are the ability to use total nucleic acids (TNA) extracts from infected hosts (to bypass viral purification limitations) for the direct one-step amplification of large FL-cDNAs, the possibility to inoculate plants with uncloned FL-cDNAs and the simplified cloning of these large molecules.

**Results:**

Using the 7.55 kb genome of *Apple chlorotic leaf spot trichovirus *(ACLSV) approaches allowing the rapid generation from TNA extracts of FL-cDNAs under the control of the T7 promoter and the successful inoculation of plants using *in vitro *transcripts obtained from these uncloned amplification products have been developed. We also show that the yeast homologous recombination system permits efficient cloning of FL-cDNAs and the simultaneous one-step tailoring of a ternary Yeast-*Escherichia coli-Agrobacterium tumefaciens *shuttle vector allowing efficient inoculation of both herbaceous and woody host plants by agroinfiltration.

**Conclusions:**

The fast and efficient strategies described here should have broad applications, in particular for the study of "difficult" plant viruses, such as those infecting woody hosts, and potentially for other, non plant-infecting viral agents.

## Background

Over the past 25 years, our ability to discover and characterize viral agents has steadily improved, leading to a constant flow of discovery of novel plant viruses as testified by the literature and by the constant increase in the number of viral species described in successive reports of the International Committee for the Taxonomy of Viruses [1]. The development of next generation sequencing (NGS) techniques promises to increase the rate at which novel plant viruses will be discovered in coming years [2,3]. At the same time, our ability to unambiguously establish the contribution of newly characterized viral agents to particular plant diseases has not improved. The fulfilling of Koch's postulates has been modified by L. Bos to be adapted to viruses, and represents a fundamental point in plant virology [4]. With the application of these postulates, the role of many viruses in diseases has been deciphered. But for many other plant viruses, technical problems in the identification of alternative herbaceous hosts, in purification or in experimental transmission have prevented the analysis of their contribution to particular diseases [4]. This is especially true for viruses affecting vegetatively propagated crops [5-7], which often have the added disadvantage of being frequently mixed infections [8]. Thus, for many viruses, the demonstration of their involvement in a given disease has not been completed, but has only been postulated on the basis of an association with symptomatic plants [see for example [9,10]].

One strategy to bypass the problems encountered with fulfilling of Koch's postulates involves the use of full-length cDNAs clones (FL-cDNAs) (or DNA clones in the case of DNA viruses) from which infectious RNA transcripts can be obtained *in vitro *or *in vivo *[11]. However, the construction of infectious FL-cDNAs is still often complicated and time-consuming for many reasons: the difficulty to optimize a standardized protocol for all viruses, the necessity of a perfect junction of the promoter and 5' end of the viral sequence, the difficulty to clone large cDNA molecules and the frequent instability of such clones [11].

These difficulties have largely limited the use of FL-cDNAs to studies on reverse genetics of well characterized viruses, which have provided access to valuable information on the expression of viral genomes, their replication and mechanisms involved in the infection cycle. They also provided further insight on the functions of different viral proteins or the mechanisms of interaction between viruses and their host plant(s) or vector(s).

However, despite their potential to address such questions, the use of infectious FL-cDNAs to confirm or refute etiological hypotheses has been rather limited [12-15]. In a recent example, the construction of an agroinfiltrable FL-cDNA clone of *Citrus leaf blotch virus *(CLBV) allowed the demonstration that CLBV is the causal agent of the Dweet mottle disease and that in single infections it does not cause the bud union crease disease [16]. An example of the widespread use of infectious constructs for etiology studies is in the *Geminiviridae *family, for which efficient techniques exist for the development of both cloned or uncloned infectious DNA constructs [17,18]. However, there are additional technical difficulties when working with RNA viruses that are responsible for the limited use of FL-cDNAs in etiology studies of RNA plant viruses.

Simplified strategies for the easier and faster development of infectious FL-cDNA for etiology studies of plant viruses should have a number of desirable properties. First, is the ability to use total nucleic acids (TNA) extracts from infected plants as template for cDNA synthesis [12,19], rather than purified viral RNA as this would bypass the need to propagate and purify the virus. Second, is the ability to use long distance PCR [20] to amplify the viral genomes as single, large PCR fragments, a technique that has been used rarely for genomes longer than 7 kb [12,19,21-23]. In a number of situations, cloning of the infectious FL-cDNA may not be necessary to validate an etiology hypothesis, so that the ability to infect plants using uncloned PCR products is also of potential interest [24-26]. Lastly, when cloning of FL-cDNAs is used, techniques that facilitate the cloning of long PCR fragments or the one-step assembly of complex constructions would be of great interest. One little used strategy with such a potential is the use of the efficient homologous recombination machinery of the yeast *Saccharomyces cerevisiae*. Until recently, the application of this system has been limited to yeast genetics and to the construction of plasmids and yeast artificial chromosomes (YACs) [27]. The full power of this approach has been demonstrated recently by the assembly of 25 overlapping DNA fragments to generate a synthetic mycoplasma genome in a single step [28]. In virology, the application of this strategy has been used as an alternative to difficult classical cloning in *Escherichia coli*, as in the case of Dengue virus type 2, where three cDNA fragments of the virus were assembled by homologous recombination in yeast to generate an infectious FL-cDNA [29]. The fact that recombination is very efficient, even with short, 20-30 nucleotides-long overlap regions between fragments created using PCR primers has facilitated the construction of recombinant viral genomes [30,31].

In the present study, *Apple chlorotic leaf spot virus *(ACLSV), the type species of the genus *Trichovirus *within the family *Betaflexiviridae *[1,32,33], was used as a model system for the development of approaches that fulfill some of the above criteria for improved preparation of infectious FL-cDNAs. The genomic RNA of ACLSV is about 7.55 kb in length [34,35] and an infectious FL-cDNA for a Japanese isolate from apple (P-205) under the control of the CaMV 35S promoter has been constructed [36]. We report on the long distance PCR amplification from TNA extracts of infectious FL-cDNAs under the control of the T7 promoter. We also show that the yeast homologous recombination system permitted the efficient cloning of such large FL-cDNAs and the simultaneous one-step tailoring of a ternary yeast-*E. coli-Agrobacterium tumefaciens *shuttle vectors allowing efficient infection of plants by agroinfiltration.

## Results

### Long distance RT-PCR amplification of ACLSV FL-cDNAs under the control of the T7 promoter and infectivity in various hosts of the transcripts synthesized *in vitro *from the uncloned PCR products

**Figure 1:**
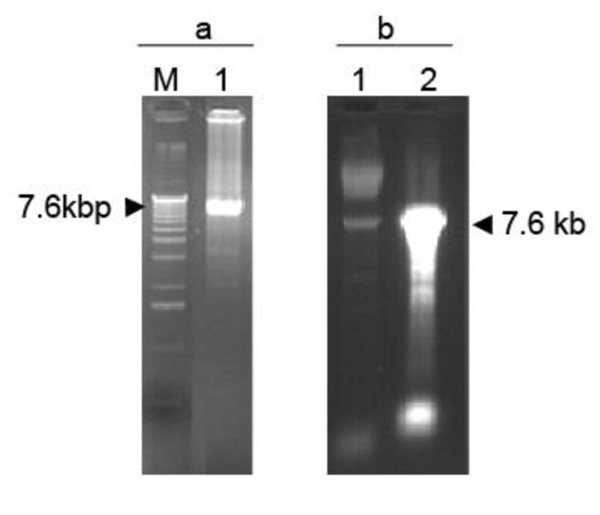
**Agarose gel electrophoresis analysis of ACLSV full length amplification products and of *in vitro *transcription products derived from them**. (a) Analysis on a 0.8% non-denaturing agarose gel of the T7-FL-cDNA LD-RT-PCR amplification product (lane 1) and of molecular mass markers (Invitrogen 1 kb ladder, lane M); (b) Analysis on a 1% denaturing agarose gel of the T7-FL-cDNA transcription products (lane 2) compared with the input purified T7-FL-cDNA LD-RT-PCR amplification product (lane 1).

The feasibility of amplification of ACLSV FL-cDNAs under the control of the T7 promoter from crude nucleic acids extracts obtained from infected plants was evaluated. The amplification was performed using primers FLAC3 and FLAC5 (Table 1), the later integrating a 18 bp sequence corresponding to the T7 promoter, including the G corresponding to the transcription start site, fused to the 25 nt 5' terminal ACLSV genomic sequence. As a consequence, transcripts synthesized on a PCR product integrating this primer are expected to have a single 5' extra G as compared to the wild type viral sequence. A good yield of a PCR product of the expected ~7.6 kbp size was obtained will all three polymerases evaluated (Figure 1a and results not shown) but amplification results using the Phusion^® ^High Fidelity DNA Polymerase (Finnzyme) proved somewhat erratic and difficult to reproduce. Both yield of the correct size product and limitation of unwanted, shorter products were improved by using 10-fold diluted first-strand cDNAs (Result not shown).

**Table 1:** Primers used for the amplification of either ACLSV FL-cDNA or the different PCR fragments used for ACLSV FL-cDNA cloning by homologous recombination in *Saccharomyces cerevisiae*

Primer name	Sequence 5'-3'^a^	Amplified fragment	Size (kbp)
FLAC5 FLAC3	TAATACGACTCACTATAG**TGATACTGATACAGTGTACACTCACG** **T_(30)_GTAGTAAAATATTTAAAAGTCTACAG**	T7-FL-cDNA^b^	7.5

30ANotvec T7ACR	**A_(30)_**GCGGCCGCTCTAGCTAGAAGCTTTTGTTCCCTTTAGTG **GTATCAGTATCA**CTATAGTGAGTCGTATTAAGATCGGACCCTGGCGTAATAGC	Yeast-pBS70T^b^	5.2

ACLSVF FLAC3	**TGATACTGATACAGTGTACACTCACGTCGTGAG** **T_(30)_GTAGTAAAATATTTAAAAGTCTACAG**	FL-cDNA^c^	7.5

30ACNOSF AC35SR	TAAATATTTTACTACA(30)CGGGTACCGAGC **CGACGTGAGTGTACACTGTATCAGTATCA**CCTCTCCAAATGAAATGAACTTCCTTATA	Yeast-pBS70T^c^	5.2

ACPCR1F FLAC3	AGAGG**TGATACTGATACAGTGTACACTCACG** **T_(30)_GTAGTAAAATATTTAAAAGTCTACAG**	FL-cDNA (PCR1)^d^	7.5

ACPCR2F PCR2	**ACA_(30)_**GAGCTCGAATTCGCTGAAATCACC TCGAGTCGTATCGGGCTACCTAGCA	Fragment of pBIN61 (PCR2)^d^	10.7

PCR3F PCR3R	TGCTAGGTAGCCCGATACGACTCGAGGGGGTGGAGCTTCCCATTGCG ATCAACTCGAGTCGGTCGAAAAAAG	Fragment of Yeast-pBS70T (PCR3)^d^	2

PCR4F ACPCR4R	CTTTTTTCGACCGACTCGAGTTGATGGCGGTCCTGGGGGCTA **TGTACACTGTATCAGTATCA**CCTCTCCAAATGAAATGAACTT	Fragment of Yeast-pBS70T (PCR4)^d^	2

LEV-R AC-F2	CGGCTCGTATGTTGTGTGGA **TTTCTACTACGCCTGAAGTGG**	Junction between 35S promoter and ACLSV FL-cDNA	0.5

a: The regions of homology introduced in the various primers to allow homologous recombination between fragments are underlined while ACLSV sequences are indicated in bold.

b: The amplified fragments have been used for cloning the ACLSV T7-FL-cDNA in Yeast-pBS70T vector

c: The amplified fragments have been used for cloning ACLSV 70S-FL-cDNA in Yeast-pBS70T shuttle vector

d: The amplified fragments have been used for cloning ACLSV 35S-FL-cDNA in ternary Yeast-*E. coli-A. tumefaciens *shuttle vector

Full length RNA molecules of ~7.6 kbp were transcribed readily *in vitro *using the T7 RNA polymerase and the purified RT-PCR amplicons (Figure 1b). A high yield of transcripts, up to 30 μg from about 200 ng of input DNA template, was obtained, even in the presence of a cap analog to insure the synthesis of capped transcripts. As shown in Table 2, the obtained transcripts were infectious when rub-inoculated to young *C. quinoa *plants. The possibility that the observed infectivity might result from ACLSV genomic RNA carried over from the TNA extract used as template for the first-strand cDNA synthesis was ruled out by digesting the FL-cDNA PCR product with *Kpn*I before the *in vitro *transcription reaction and inoculating the resulting truncated transcripts, which never gave rise to plant infection (Result not shown). The transcripts obtained from PCR products generated using the high fidelity Phusion^® ^polymerase seem to have a higher infectivity than those prepared using the Advantage^® ^GC Genomic LA Polymerase Mix. Unfortunately, amplification using the Phusion^® ^enzyme was far less reproducible than with the lower fidelity enzyme mix. In *C. quinoa*, the transcripts were also found to be infectious upon biolistic inoculation (Results not shown) but because of the cost of this technique and of the fact that it did not seem to bring about an improvement in inoculation efficiency over mechanical inoculation, this was not pursued further.

**Table 2:** Infection rates of two ACLSV hosts following mechanical inoculation of *in vitro *transcripts obtained from ACLSV FL-cDNA under the T7 promoter synthesized using two different PCR enzymes

Enzyme used for LD-PCR	Infected/inoculated plants (% infected)^a,b^	
	
	Chenopodium quinoa	Nicotiana occidentalis 37B
Advantage GC^c^	8/36 (22%)	0/22 (0%)
Phusion^d^	30/36 (83%)	0/22 (0%)
water inoculation control^e^	0/12 (0%)	0/12 (0%)

a: the results shown are the sum of two inoculation experiments performed with transcripts deriving from PCR products resulting from different amplification reactions.

b: plants were mechanically inoculated using 5 μg of transcripts per plant.

c: Advantage^® ^GC Genomic LA Polymerase Mix (Clontech)

d: Phusion^® ^High Fidelity DNA Polymerase (Finnzyme)

e: plants were rub inoculated using the DEPC-treated sterile water used to resuspend the *in vitro *transcripts.

Surprisingly, inoculation of another herbaceous host of ACLSV, *Nicotiana occidentalis *37B, never gave rise to infection despite the fact that high infection rates were observed in *C. quinoa *using the same batches of transcripts in parallel experiments (Table 2). The transcripts were also inoculated to ACLSV woody hosts (GF305 peach or apple), either by mechanical (stem slashing of young GF305 peach seedlings) or biolistic inoculation (germinating GF305 peach or apple seedlings), but none of the plants thus treated developed ACLSV infection (Result not shown).

### One-step cloning by homologous recombination in yeast cells of ACLSV FL-cDNAs

**Figure 2:**
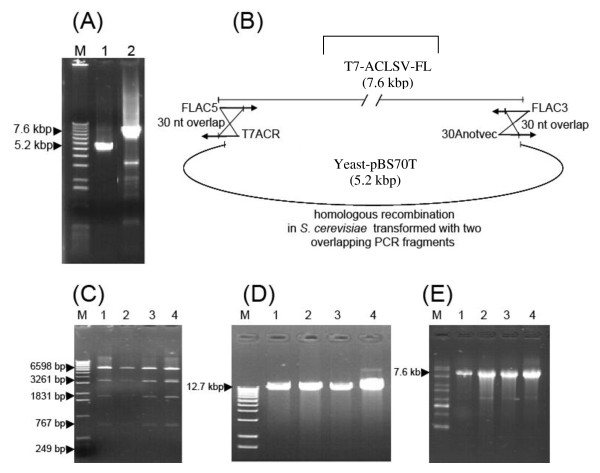
**One-step cloning by homologous recombination in yeast of an ACLSV FL-cDNA under the control of T7 promoter**. (A) 0.8% non-denaturing agarose gel electrophoresis of the two PCR fragments used to transform yeast cells. Lane 1: PCR product corresponding to the Yeast-PBS70T amplified using the divergent T7ACR/30Anotvec primer pair; Lane 2: ACLSV T7-FL-cDNA amplified using the FLAC5/FLAC3 primer pair; (M): Molecular mass marker (Invitrogen 1 kb ladder). (B) Schematic representation of the cloning by homologous recombination strategy. The 30 bp overlapping regions between the two PCR fragments in which homologous recombination takes place are indicated. (C) *Bgl*II restriction analysis (lanes 1-4) of four pools of 10 independent recombinant plasmids recovered following retransformation of *E. coli *cells using the plasmid preparation purified from the bulked transformant yeast cells, (M): Molecular mass markers (Invitrogen1 kb ladder). (D) Analysis by non-denaturing 1% agarose gel electrophoresis of the same four pools of plasmids, following linearization with *Not*I (lanes 1-4), (M): Molecular mass markers (Invitrogen 1 kb ladder). (E) Analysis by 1% denaturing agarose gel electrophoresis of the *in vitro *RNA transcripts synthesized from the *Not*I-linearized plasmid pools, (M): 0.5-10 kb RNA ladder (Sigma).

Although uncloned infectious FL-cDNAs under the control of either the T7 or the CaMV 35S promoters might be very useful for the validation of many etiological hypotheses, cloned infectious cDNAs are also of great potential interest. However, the cloning of large PCR products often shows a very low efficiency and thus it was decided to utilize the efficient homologous recombination properties of the yeast *S. cerevisiae *to generate FL-cDNAs. Experiments were conducted in parallel using either a T7 FL-cDNA obtained as described above or a FL-cDNA without added terminal sequences amplified using the ACLSVF/FLAC3 primer pair (Table 1). Figure 2B illustrates the strategy of cloning of the T7 FL-cDNA in the shuttle vector Yeast-pBS70T.

The receiving Yeast-pBS70T vector was amplified using the divergent 30ANotvec/T7ACR or 30ACNOSF/AC35SR primer pairs (Table 1 and Figure 2) to generate a linear (~5.2 kbp) form of the plasmid having 30 bp regions of homology with the ACLSV T7 FL-cDNA or FL-cDNA to be cloned, respectively. Yeast cells were then transformed with equimolar amounts of the two PCR products (linear plasmid and ACLSV FL-cDNA to be cloned). Homologous recombination between the two linear molecules produces a circular plasmid which can replicate and allow the expression of the *TRP1 *selection marker. Yeast cells harboring recombinant plasmids were pooled, grown under selection pressure and a pool of recombinant plasmids extracted from these yeast cells used to transform *E. coli *cells by electroporation. For each cloning experiment, recombinant plasmids from 94 *E. coli *colonies were checked by directly amplifying the junction between the vector and the ACLSV FL-cDNA using primer pair LEV-R/AC-F2 (Table 1). For the cloning of the FL-cDNA under the T7 promoter, 60 colonies (64%) gave a positive amplification signal while the corresponding value was 49 colonies (52%) for the cloning under the 70S promoter.

The plasmids harbored by the colonies giving a positive amplification were then extracted and submitted to restriction enzyme mapping (Figure 2C). In total, 40 and 30 recombinant plasmids had the expected *Bgl*II restriction pattern and were considered as candidates for the cloned T7-cDNA and 70S-cDNA, respectively. Pools of 10 such plasmids were then prepared and their infectivity assayed by mechanical inoculation of *in vitro *transcripts prepared from the *Not*I-linearized plasmids pools (T7-cDNA, Figure 2D &2E) or by biolistic inoculation of the plasmid pools themselves (70S-cDNA). For pools showing infectivity, the infectivity of the 10 pooled plasmids was then individually evaluated.

The results obtained are presented in Table 3. In the case of the ACLSV FL-cDNA cloned under the control of the T7 promoter (T7-cDNA), a single pool (Pool 3) showed infectivity and deconvolution of that pool allowed the identification of a single plasmid from which infectious *in vitro *transcripts could be produced. As expected from the experiments with the uncloned T7-cDNA products, no infectivity was observed upon inoculation of the transcripts derived from this recombinant plasmid to *N. occidentalis *37B (Result not shown).

**Table 3:** Infectivity on *Chenopodium quinoa *plants and number of infectious plasmids in plasmid pools obtained upon cloning by homologous recombination in yeast cells of ACLSV FL-cDNA under the control of the T7 promoter (T7-cDNA) or of the duplicated 35S promoter (70S-cDNA)

Plasmid pools	Infected/inoculated *C. quinoa *plants^a^	Infectious plasmids per pool
T7-cDNA Pool 1	0/10	0
T7-cDNA Pool 2	0/26	0
T7-cDNA Pool 3	10/16	1
T7-cDNA Pool 4	0/26	0

70S-cDNA Pool 1	5/6	1
70S-cDNA Pool 2	4/6	1
70S-cDNA Pool 3	0/12	0

a: 3 μg of capped transcripts per *C. quinoa *plant were used for inoculation of transcripts from T7-cDNA plasmid pools while about 160 ng of plasmids were used per plant for biolistic inoculation of 70S-cDNA pools (2 shots per plant).

In the case of the ACLSV FL-cDNA cloned under the control of the 70S promoter (70S-cDNA), two of the 3 tested pools led to infection of the inoculated plants (Pools 1 and 2) and, upon deconvolution, each pool was shown to contain a single infectious plasmid (Table 3). For the two ACLSV FL-cDNA cloning attempts the rate of recovery of infectious clones is therefore of about 1-2%.

### One-step cloning of ACLSV FL-cDNA and construction of a ternary Yeast*-E. coli-A. tumefaciens *shuttle vector to generate an agroinoculable ACLSV infectious cDNA clone

**Figure 3:**
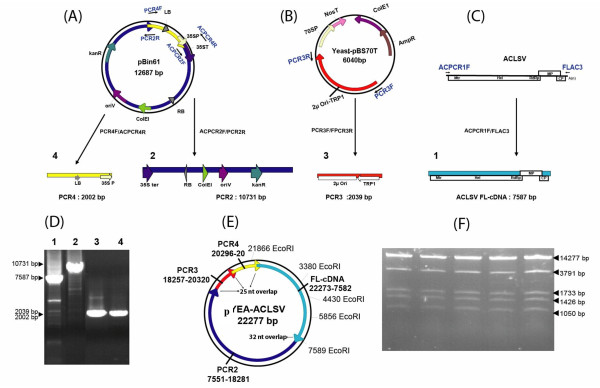
**One-step assembly by homologous recombination in yeast of a ternary Yeast-*Escherichia coli*-*Agrobacterium tumefaciens *shuttle vector and simultaneous cloning of an ACLSV FL-cDNA to generate an infectious agroinoculable full-length viral cDNA clone**. (A, B, C) Schematic representation of the strategy used to generate the four PCR fragments from which the construct is assembled by homologous recombination. The primers used are indicated by blue arrows near the respective PCR templates and are listed in Table 1. Regulatory elements are indicated by arrows of various colors. LB: T-DNA left border; 35S P: CaMV 35S promoter; 35S ter: CaMV 35S terminator; RB: T-DNA right border; ColE1: *E. coli *ColE1 origin of replication; oriV: *A. tumefaciens *oriV origin of replication; KanR: kanamycin resistance gene; 2μ Ori: yeast 2μ origin of replication; TRP1: yeast *TRP1 *selection gene. (A) Map of pBin61 from which are amplified the ~10.7 kbp fragment (PCR2, dark blue, carrying the 35S ter, the RB, the ColE1 and OriV replication signals and the KanR selection marker) and the ~2 kbp fragment (PCR4, yellow, carrying the LB and the 35S promoter) using respectively primer pairs ACPCR2F/PCR2R and PCR4F/ACPCR4R. (B) Map of Yeast-pBS70T used to amplify the ~2 kbp fragment (PCR3, red, carrying the yeast 2μ Ori-TRP1) using the PCR3F/PCR3R primer pair. (C) Genomic organization of ACLSV and position of the ACPCR1F/FLAC3 primer pair used to amplify the FL-viral cDNA (PCR1, light blue). (D) Non-denaturing 0.8% agarose gel electrophoresis of the four purified overlapping PCR products. (E) Schematic representation of the recombinant YEA-ACLSV ternary plasmid carrying the ACLSV FL-cDNA obtained by assembly by homologous recombination of the four fragments. The position of *Eco*RI sites used for restriction mapping of the plasmid is shown. (F) Non-denaturing 0.8% agarose gel electrophoresis of 5 independently obtained recombinant plasmids showing the expected *Eco*RI digestion pattern.

The use of infectious FL-cDNA constructs that can be inoculated by agroinfection or by agroinfiltration often results in a higher rate of infected plants or may allow the successful inoculation of hosts that could not be infected upon mechanical or biolistic inoculation [see for example [16]]. We therefore decided to try to obtain an infectious agroinoculable ACLSV FL-cDNA construct. This was achieved in a single step of simultaneously assembling a ternary Yeast-*E. coli-A. tumefaciens *shuttle vector and cloning the ACLSV FL-cDNA in this vector. For this purpose, four overlapping PCR fragments were assembled by homologous recombination in yeast (Figure 3). Following transfer to *E. coli *of the recombinant plasmids isolated from yeast cells growing under selection, restriction mapping with *Eco*RI showed that 36 of the 40 tested *E. coli *colonies contained a plasmid with the expected restriction pattern (Figure 3E and 3F). These 36 plasmids were pooled into three pools of 12 plasmids and the infectivity of these three pools was evaluated. Given that the ACLSV FL-cDNA is cloned in the ternary vector under the control of the 35S promoter, this construct is expected to be infectious either as purified plasmid inoculated by biolistic bombardment or by agroinoculation of *A. tumefaciens *cells harboring it. Both inoculation strategies were therefore evaluated for each pool of recombinant plasmids: biolistic inoculation of *C. quinoa *plants or agroinfiltration of *C. quinoa *or *N. occidentalis *37B plants. The results are presented in Table 4.

**Table 4:** Infectivity of pools of agroinoculable ternary Yeast-*Escherichia coli-Agrobacterium tumefaciens *shuttle vectors containing the ACLSV FL-cDNA under the control of the 35S promoter assembled by one-step homologous recombination-based cloning in yeast

Plasmid pools^a^	Infected/biolistically inoculated^b^	Infected/Agroinoculated^c^		Infectious plasmids per pool
			
	C. quinoa	C. quinoa	N. occidentalis 37B	
1	0/6	0/6	0/5	0
2	0/6	0/6	0/5	0
3	3/5	4/6	2/4	1

a: each pool was composed of 12 recombinant plasmids showing the expected restriction mapping results

b: biolistic inoculation was performed using two shots par plant and 100 ng of the pool of purified plasmids per shot (about 8 ng of individual plasmid per shot).

c: Agroinoculation was performed by infiltrating two leaves of each plant with the suspension of agrobacterium cells.

Of the three pools tested, one gave successful inoculation of *C. quinoa *plants by either of the inoculation techniques used or of *N. occidentalis *37B plants by agroinfiltration. Deconvolution of this pool allowed the identification of a single infectious recombinant ternary vector (pYEA-ACLSV). Biolistic bombardment of *C. quinoa *with purified YEA-ACLSV resulted in a 60% infection rate, which is to be compared with the 90% infection rate observed using plasmid pCLSF under similar inoculation conditions (2 shots per plant, 50 ng purified plasmid per plant). Inoculation by agroinfiltration with *A. tumefaciens *cells carrying pYEA-ACLSV resulted in infection rates of 91% and 67% for *C. quinoa *(a total of 33 inoculated plants in 3 experiments) or *N. occidentalis *37B (a total of 18 inoculated plants in 3 experiments), respectively.

Inoculation by agroinfiltration of pYEA-ACLSV to young GF305 peach seedlings demonstrated that this construct was also able to generate infection in this woody host since 2 out of the 7 inoculated plants were later shown to be infected (result not shown).

As a control, a FL-cDNA was amplified directly from pCLSF and similarly cloned in a ternary Yeast-*E. coli-A. tumefaciens *shuttle vector. Out of 64 analyzed recombinant plasmids, 50 had the expected *Eco*RI restriction pattern and were divided into 5 pools of 10 plasmids. All pools proved infectious when inoculated either by biolistic or by agroinfiltration, in either *C. quinoa *or *N. occidentalis*. Individual testing of 3 randomly selected plasmids from each of those five pools revealed all of them to be infectious (Results not shown).

## Discussion

Long Distance (LD) RT-PCR amplification had been used successfully to generate infectious uncloned PCR products [24,26] but starting with purified viral RNA preparations obtained from purified viral particles. The need for such purified viral RNA preparations constitutes a severe limitation, in particular for viruses infecting woody hosts and for which alternative herbaceous hosts and/or purification techniques may not be available. Although the results presented here were obtained with total extracts from *C. quinoa*, recently the same technique was used successfully to amplify the complete genome of several isolates of *Apricot latent virus *(ApLV, 9.2-9.3 kb) from total RNA extracts obtained from infected GF305 peach seedlings, which indicates that the protocol developed is not limited to herbaceous hosts extracts or to the 7.5 kb length of the ACLSV genome (F. Youssef *et al*., unpublished). On the other hand, it was not possible to obtain similar results when using viral double-stranded RNA (dsRNAs) preparations or immunocaptured virions. Given the infectivity of the pYEA-ACLSV agroinoculable construct to GF305 peach seedlings, all the steps seem today at hand for going from TNA extracts obtained from woody hosts to successful inoculation of such woody hosts using uncloned or cloned constructs. The efficiency of such a process remains, however, to be directly evaluated. Similarly, whether the presence in the original host of several viral strains in mixed infection (as often observed in woody hosts) will reduce the efficiency of production of infectious constructs remains to be evaluated.

All three evaluated enzymes (Advantage^® ^GC Genomic LA Polymerase Mix, Expand High Fidelity PCR System and Phusion^® ^High Fidelity DNA Polymerase) allowed, although with variable efficiency and reproducibility, the amplification of the T7-FL-cDNA. The results obtained in the present work indicate that the inoculation efficiency may vary considerably depending on the inoculated host species. While such an observation is not overly remarkable when comparing the inoculation of herbaceous hosts with that of the notoriously more difficult woody hosts, the large difference observed when comparing mechanical (Table 2) or biolistic (results not shown) inoculation of *in vitro *transcripts on *C. quinoa *and on *N. occidentalis 37B *is more surprising. Several hypotheses can be proposed to explain this observation, including an intrinsic difference in the susceptibility of these two host plants, possibly linked with a more rapid degradation of inoculated transcripts in *N. occidentalis *or, on the contrary, the need for host-specific adaptative mutations in ACLSV in order to infect this host. The fact that, contrary to the situation in *C. quinoa*, a significant proportion of *N. occidentalis *plants sometimes escape infection upon mechanical inoculation with crude plant homogenates, even when the viral isolate used had been passaged in this host argues against the second hypothesis. However, further experiments are required to clarify this point that could have important practical implications in view of the fact that *N. occidentalis *is the only herbaceous host known for many fruit tree-infecting *Betaflexiviridae*. In this respect, conflicting results were reported previously, since Saldarelli *et al. *[26] were able to infect this host with transcripts of the *Grapevine virus A *and *Grapevine virus B *vitiviruses while Vives *et al. *[16] could only obtain *Citrus leaf blotch virus *infection when using agroinoculation of a full-length construct.

Although PCR-amplified viral FL-cDNA molecules have been cloned with success in *E. coli *vectors [for example [12,13,16,19,21-23]], such cloning frequently is difficult, in particular for large FL-cDNAs. We therefore evaluated the potential usefulness of the yeast homologous recombination system for the one-step cloning of the ACLSV FL-cDNAs. As demonstrated by the successful simultaneous assembly of the ternary shuttle vector and cloning of the ACLSV FL-cDNA, this system is indeed versatile and efficient. Given that it has been used for the simultaneous assembly of numerous large DNA fragments [28], it should not prove particularly sensitive to the size of the cloned viral genome. However, in the experiments reported here, infectious plasmids only represented a fraction of all plasmids that showed the expected pattern upon restriction mapping (from 2.5% to 6.5% depending on experiment). A similarly low rate of recovery of infectious cDNA clones was reported for another *Betaflexiviridae*, CLBV [16]. Spetz *et al. *[19] reported a very low recovery of infectious constructs and the only recovered plasmid showed different biological properties than the input virus. In the experiments reported here, the rate of recovery of infectious constructs was dramatically improved when a FL-cDNA amplified from an infectious cDNA clone was used instead of one amplified from total RNAs from infected plants. The low rate of recovery of infectious constructs might be due to either a low rate of infectious molecules in the starting viral RNA population and/or the introduction of detrimental mutations by the reverse transcription step but seem to exclude an impact of the PCR amplification step. In any case, the pooling strategy reported here allowed the efficient and rapid identification of the few infectious clones. It could probably be further improved by using a two or three dimensional pooling approach, so that individual infectious molecules could be identified in a single step. The potential to assemble multiple DNA fragments [28] could also be exploited to join in a single step partial cDNA fragments spanning the very long genomes [29] of viruses such as members of the *Closteroviridae *family.

## Conclusions

We report here for the first time a set of techniques and amplification conditions that allow the use of the crude total RNA extracts from ACLSV infected *C. quinoa *plants as templates for the Long Distance (LD) RT-PCR amplification of infectious full-length viral cDNAs. As far as we can ascertain, this represents the largest RNA plant virus genome amplified by PCR from TNA extracts. We also demonstrate that homologous recombination in yeast allows for the fast and efficient cloning of infectious FL-cDNAs and/or one-step tailoring of complex constructs.

Overall the strategies reported here allow for the rapid generation of uncloned or cloned infectious FL-cDNA from total RNA preparations from infected hosts and should prove useful in a range of studies, and, in particular, for the validation of etiological hypotheses involving difficult to manipulate plant viral agents.

## Materials and methods

### Virus source and RNA extraction

The P-205 Japanese strain of ACLSV, [34], was provided by Dr. N. Yoshikawa (Iwate University, Japan) as an infectious cDNA clone under the control of the CaMV 35S promoter, pCLSF [36]. The viral isolate obtained upon biolistic inoculation of pCLSF to *C. quinoa *was propagated in this host by mechanical inoculation and used as source of virus or of viral nucleic acids. Total nucleic acids were extracted with the SV Total RNA Isolation System (Promega, Lyon, France) from 30 mg of leaf tissue from symptomatic *C. quinoa *plants, following the manufacturer's instructions. The yield was approximately 15 μg of RNAs in 60 μl of sterile water.

### Full-length ACLSV cDNA amplification using Long Distance (LD) RT-PCR

Synthesis of the first-strand cDNA was primed with oligonucleotide FLAC3: 5' T(30)GTAGTAAAATATTTAAAAGTCTACAG 3', which is complementary to the 3'-terminal nucleotides (positions 7552-7527) of the genomic RNA of ACLSV-P205 (GenBank D14996). The synthesis was performed using about 1 μg of TNA and the PrimeScript™ Reverse Transcriptase (Clontech/TaKaRa Bio Europe, Saint-Germain en Laye, France) or the Expand Reverse Transcriptase (Roche Diagnostics, Meylan, France), following the manufacturer instructions. In order to obtain a FL-cDNA under the control of the T7 promoter, primer FLAC5 (5' ***TAATACGACTCACTATAG***TGATACTGATACAGTGTACACTCACG 3') was used in combination with FLAC3 in a LD-PCR experiment. FLAC5 contains the T7 promoter (in bold and italic) and the first 26 5' nucleotides of the genome of ACLSV-P205. Three commercial DNA polymerases or DNA polymerases mixes were compared for their efficiency in this study: the Advantage^® ^GC Genomic LA Polymerase Mix (Clontech), the Expand High Fidelity PCR System (Roche) and the Phusion^® ^High Fidelity DNA Polymerase (Finnzyme/Fischer Scientific, Illkirch, France). All enzymes were used according to their supplier recommendations, using as template 3 μl from the first strand cDNA product either undiluted or diluted 10-fold. All PCRs were performed in a 25 μl final reaction volume. The PCR cycling parameters recommended by the suppliers were used, with the exception of the annealing temperature which was fixed at 60°C for all experiments. Amplicons were purified using the MSB^® ^Spin PCRapace kit (Invitek, les Ullis, France) and eluted in Diethylpyrocarbonate (DEPC) treated, distilled water. They were then quantified by electrophoresis on non-denaturing agarose gels and image analysis using the Image J 1.42q software (W. Rasband, NIH, USA). As a control in some experiments, LD-PCR amplification was performed directly on the pCLSF infectious plasmid instead of on cDNAs obtained from TNA extracts.

### T7 RNA polymerase in vitro transcription of ACLSV FL-cDNAs

Two hundred nanograms of purified ACLSV FL-cDNA amplicons were used in *in vitro *transcription reactions performed using the phage T7 RNA polymerase and the mMESSAGE mMACHINE kit (Ambion, Courtaboeuf, France) in the presence of the cap analogue m^7^G(5')ppp(5')G (Ambion). At the end of the synthesis, which was performed as recommended by the kit supplier, transcripts were treated with 1 μl of TURBO DNase (Ambion) and then used either directly or after purification on Macro SpinColumn™G-50 (Harvard Apparatus, Les Ullis, France) following the manufacturer's instructions. Transcripts were quantified as described above for the PCR products and, if necessary, were stored at -80°C until use in plant inoculation assays.

### Inoculation of herbaceous or woody ACLSV host plants

The ACLSV FL-RNA transcripts were inoculated on young (four leaf stage) plants of *C. quinoa *or *N. occidentalis *cv. 37B by gently rubbing 5 μl of *in vitro *transcripts (adjusted to the desired concentration with DEPC-treated distilled water) on two celite-dusted leaves of each plant (Celite 545, 0,01-0,04 mm, Merck/VWR, Fontenay-sous-Bois, France). Following inoculation, leaves were rinsed briefly with distilled water. As control, plants were inoculated using DEPC-treated distilled water without RNA transcripts. All plants were grown (16 hours day/8 hours night) and observed weekly for symptoms development.

Alternatively, the ACLSV FL-RNA transcripts were biolistically inoculated on leaves of young *C. quinoa *plants using the Helios Gene Gun (Bio-Rad/Marnes-La-Coquette, France). For inoculation, 5 μg of transcripts were used to prepare 10 cartridges as recommended by Bio-Rad, using 1 μm gold particles. Each plant was bombarded on two leaves, using a 200 psi pressure. For the inoculation of woody host plants, young germinating peach (*Prunus persicae*, cv. GF305) or apple (*Malus domestica*) seedlings were inoculated when the growing radicle had reached a length of about 0.5-1 cm. Briefly, the envelopes of germinating seeds were gently removed and the cotyledons were then inoculated by biolistic bombardment (1 shot on each of the cotyledons for the GF305 and 3 shots for groups of 10 germinating seeds for *M. domestica*. After inoculation, seeds were rinsed with distilled water and then placed on wet tissue paper in Petri dishes at 4°C in the dark for 24 hours. The next day, they were treated by a broad spectrum fungicide by soaking for 30 min in a 0.5% solution of Propamocarb-HCl followed by rinsing with water. The germinating embryos were finally transplanted to sterile moist sand and left to develop in the greenhouse up to the 4-leaf stage before being transplanted into pots containing potting soil. As control, plants were biolistically inoculated using the infectious pCLSF plasmid.

### ACLSV detection in inoculated plants

ACLSV was detected as described [37], using double-antibody sandwich enzyme-linked immunosorbent assay (DAS-ELISA) and immunoglobulins from the P2 polyclonal antiserum raised against the P863 ACLSV isolate (INRA, Bordeaux, France). Alternatively, ACLSV infection was detected using the A52-A53 ACLSV-specific RT-PCR assay described [38]. Identity of the amplified virus was confirmed by direct sequencing of the amplification products (Beckman Coulter Genomics, Meylan, France).

### One step cloning of ACLSV FL-cDNAs by homologous recombination in *Saccharomyces cerevisiae*

The yeast (*S. cerevisiae*) strain YPH501 (*MAT*a/*MAT*α *ura3-52 lys2-801*amber *ade2-101*ochre *trp1-*Δ*63 his3-*Δ*200 leu2-*Δ*1*) was used [39]. The yeast-*E. coli *vector used in these experiments is a pBS70T plasmid [40] in which a DNA fragment containing a yeast 2μ origin of replication and the yeast *TRP1 *gene as a selection marker has been inserted (Yeast-pBS70T). The pBS70T backbone provides an *E. coli *ColE1 origin of replication and an ampicillin resistance gene for selection in *E. coli*, in addition to signals for transcription *in planta*: duplicated CaMV 35S promoter (70SP) and Nopaline synthase gene terminator (NosT) of the cloned FL-cDNA. The complete vector was amplified using the Phusion^® ^High Fidelity DNA Polymerase (Finnzyme) and either one of the divergent primer pairs shown in Table 1. This strategy produces PCR products that represent a linear copy of the vector with terminal regions of 30 nt overlapping with the ends of the FL-cDNA PCR product to be cloned. All PCR products were purified using the MSB^® ^Spin PCRapace kit before use.

Yeast cultures were grown at 30°C in YPD medium (1% yeast extract, 2% peptone, 2% glucose) prior to transformation and in SD (Minimal synthetic defined Base) selective medium with the mixture of the amino acid without tryptophan (-Try DO, Dropout) after transformation. Yeast was transformed using a lithium acetate method and denatured carrier DNA as described [41], following the modifications of [30]. Approximately 1-2 μg in total of the mixture of the PCR fragments in equimolar amounts were used per transformation. All yeast colonies growing on the selection plates following transformation were collected in 3 ml of SD liquid medium and grown for 12 h at 30°C. Plasmid DNA was isolated from these cultures according to [42] and used for transformation of *E. coli *cells (XL1) by electroporation. Further characterization of the recombinant plasmids and their large-scale purification were carried out using standard protocols [43]. All junctions in the recombinant plasmids obtained were confirmed by sequencing (Beckman Coulter Genomics).

### One step construction of an agroinoculable ACLSV FL-cDNA in a ternary yeast-E. coli-A. tumefaciens vector

ACLSV FL-cDNA was cloned in a *de novo *constructed ternary yeast-*E. coli*-*A. tumefaciens *vector by homologous recombination in yeast cells. This was achieved by simultaneous transformation of yeast cells using 4 PCR products having 25-32 bp overlapping ends derived from the PCR primers (Figure 3 and Table 1). Besides the ACLSV FL-cDNA, the three fragments used were: (1) a 10.7 kbp product covering the 35S terminator, the T-DNA right border (RB), the *E. coli *ColE1 origin of replication, the *A. tumefaciens *OriV origin of replication and finally the kanamycin resistance gene from the pBin61 binary vector [44], (2) a ~2 kbp fragment containing the yeast 2μ origin of replication and the yeast *TRP1 *gene derived from the Yeast-pBS70T vector described above and (3) a ~2 kbp fragment containing the T-DNA left border (LB) and the CaMV 35S promoter of pBin61. The long fragment was amplified using the Advantage^® ^GC Genomic LA Polymerase Mix kit while the two shorter ones were amplified with the Phusion^® ^High Fidelity DNA Polymerase. After purification of all fragments using the MSB^® ^Spin PCRapace kit, yeast cells were transformed with a total of about 3 μg of the four fragments in equimolecular amounts. The various steps following transformation were carried out as described above and the recombinant plasmids harbored by *E. coli *colonies finally verified by restriction analysis. Plasmids showing the expected *Eco*RI restriction pattern were divided into pools and used to transform by electroporation (with ~100 ng of purified plasmid pools) *A. tumefaciens *C58C1 cells carrying the pG90 helper plasmid (provided by S. Vernhettes, INRA-Versailles). *Agrobacterium *transformants were selected on LB medium plates supplemented with 50 mg/l rifampicin, 50 mg/l kanamycin and 20 mg/l gentamycin. All transformed bacteria growing on the Petri dishes were collected and grown as pools in LB liquid medium with the same antibiotics and this culture was then used as the starter culture to prepare *Agrobacterium *cells for agroinfiltration.

### Inoculation of plants using agroinfiltration

For agroinfiltration of plants, *A. tumefaciens *cells carrying the relevant plasmid(s) were first grown overnight at 28°C in 5 ml of LB medium plus selection antibiotics (see above). These pre-cultures were then used to inoculate 25 ml cultures of induction medium [LB medium supplemented with selection antibiotics, 10 mM of 2N-morpholino-ethane sulfonic acid (MES) and 20 μM of acetosyringone]. Following incubation overnight at 28°C under agitation, bacteria were collected by centrifugation at 6000 g for 15 min at room temperature, re-suspended in infiltration medium (10 mM MgCl_2_, 10 mM MES, pH 5.6, and 150 μM acetosyringone) and the bacterial suspension adjusted to an optical density of 0.6 at 600 nm. The suspension was then incubated for 3 h at room temperature before being infiltrated in the intercellular spaces of young *C. quinoa *or *N. occidentalis *leaves, using a syringe directly placed on the lower leaf surface. Alternatively, young GF305 peach seedlings were inoculated by injections of the Agrobacterium cells suspension in their stems using a syringe and a small gauge needle. Following their inoculation, plants were monitored weekly for symptoms appearance and ACLSV infection was confirmed as described above.

## List of abbreviations

70SP: duplicated CaMV 35S promoter; ACLSV: *Apple chlorotic leaf spot virus*; ApLV: *Apricot latent virus*; CLBV: *Citrus leaf blotch virus*; DAS-ELISA: double-antibody sandwich enzyme-linked immunosorbent assay; DEPC: diethylpyrocarbonate; dsRNA: double stranded RNA; FL-cDNA: full-length infectious cDNA clone; LB: T-DNA left border; LD-PCR: long distance PCR; MES: 2N-morpholino-ethane sulfonic acid; NGS: next generation sequencing; NosT: Nopaline synthase gene terminator; RB: T-DNA right border; SD: minimal synthetic defined base; TNA: total nucleic acids; YAC: yeast artificial chromosome

## Competing interests

The authors declare that they have no competing interests.

## Authors' contributions

All authors contributed to the results presented. FY, AM, PG and TC contributed to and edited the manuscript. All authors read and approved the final manuscript.
